# Human Development Index of the maternal country of origin and its relationship with maternal near miss: A systematic review of the literature

**DOI:** 10.1186/s12884-020-02901-3

**Published:** 2020-04-16

**Authors:** Santiago García-Tizón Larroca, Francisco Amor Valera, Esther Ayuso Herrera, Ignacio Cueto Hernandez, Yolanda Cuñarro Lopez, Juan De Leon-Luis

**Affiliations:** 1 Maternal Fetal Medicine, Department of Obstetrics and Gynaecology, HGUGM, Calle O’ Donnell, 48, Planta 0, 28009 Madrid, Spain; 2 Department of Public and Maternal-Infant Health, Complutense University, Madrid, Spain

**Keywords:** Maternal near miss, Maternal mortality, Human Development Index, Immigrants

## Abstract

**Background:**

The reduction in maternal mortality worldwide has increased the interest in studying more frequent severe events such as maternal near miss. The Human Development Index is a sociodemographic country-specific variable that includes key human development indicators such as living a long and healthy life, acquiring knowledge, and enjoying a decent standard of living, allowing differentiation between countries. In a globalised environment, it is necessary to study whether the Human Development Index of each patient's country of origin can be associated with the maternal near-miss rate and thus classify the risk of maternal morbidity and mortality.

**Methods:**

A systematic review of the literature published between 2008 and 2019 was conducted, including all articles that reported data about maternal near miss in their sample of pregnant women, in addition to describing the study countries of their sample population. The Human Development Index of the study country, the maternal near-miss rate, the maternal mortality rate, and other maternal-perinatal variables related to morbidity and mortality were used.

**Results:**

After the systematic review, eighty two articles from over thirty countries were included, for a total of 3,699,697 live births, 37,191 near miss cases, and 4029 mortality cases. A statistically significant (*p* <0.05) inversely proportional relationship was observed between the Human Development Index of the study country and the maternal near-miss and mortality rates. The most common cause of maternal near miss was haemorrhage, with an overall rate of 38.5%, followed by hypertensive disorders of pregnancy (34.2%), sepsis (7.5%), and other undefined causes (20.9%).

**Conclusions:**

The Human Development Index of the maternal country of origin is a sociodemographic variable allowing differentiation and classification of the risk of maternal mortality and near miss in pregnant women. The most common cause of maternal near miss published in the literature was haemorrhage.

**Trial registration:**

PROSPERO ID: CRD 42019133464

## Background

Worldwide, over 1500 women die every day due to complications of pregnancy or childbirth. It is possible that most of these deaths could be prevented if the women were in countries other than their countries of origin. Although the Millennium Development Goal of reducing maternal mortality (MM) by 75% between 1990 and 2015 has not been achieved globally, significant progress has been made; in many countries, maternal health has improved significantly, and the goals for 2030 are to achieve MM rates of less than 70 per 100,000 live births and to increase the proportion of births attended by skilled health personnel [1]. One of the Millennium Development Goals set in 2000 by the member countries of the United Nations is to improve the health of women through multiple interventions, such as promoting access to family planning services and emergency obstetric care by qualified and trained personnel. In this respect, women in low-income countries are especially vulnerable to dying from obstetric causes. The World Health Organization, through its “Global Strategy for Women´s, Children´s and Adolescents´ Health (2016-2030),” is analysing relevant indicators and scores to improve the survival of newborns and pregnant women. Although the world has made substantial progress on these two issues, the decline in maternal and neonatal mortality has recently slowed down. Moreover, in 2017-2019, the Quality of Care Network group supported by the WHO included more countries – such as Ethiopia, Ghana, India, Malawi, Nigeria, Tanzania and Uganda – on its agenda to complete the following tasks:

- Accelerate action by adapting the WHO’s standards for improving the quality of maternal and newborn care in health facilities at the country level.
- Foster learning and generate evidence on quality of care through a learning platform.
- Develop and support institutions and mechanisms that will ensure accountability for quality of care by designing a national accountability framework.

Traditionally, the analysis of maternal deaths has been the approach of choice for evaluating women's health and the quality of obstetric care. However, due to the success of modern medicine, such deaths have become very rare in developed countries, which has led to an increased interest in analysing so-called “near miss” events. The World Health Organization defines a maternal near miss (MNM) as “a woman who nearly died but survived a complication that occurred during pregnancy, childbirth or within forty-two days of termination of pregnancy”. A MNM is also assumed to be a better indicator than MM alone when designing, monitoring, following-up and evaluating safe motherhood programmes [2]. Year after year, increasingly more authors are interested in publishing MNM events that occur in their countries, and it is necessary to analyse morbidity and mortality data over the past decade to compare situations in different countries.

Haemorrhage, hypertensive disorders of pregnancy, and infections stand out as the direct causes of more than 70% of both MNM and mortality. In all these cases, the lack of care or access to care, the high cost of health care or its poor quality, and the variation among different countries results in 1 million maternal orphans every year, and these children are also more likely to die during the years following their mother's death.

For years, gross national income per capita has been used to weigh differences among countries; however, in the 1990s, the WHO introduced the Human Development Index (HDI) as a sociodemographic variable to help differentiate countries, thus avoiding reliance on the purely economic value of each nation and trying to classify the world population in homogeneous groups through more comprehensive indicators.

This index has helped the WHO to establish different strategies to end preventable maternal morbidity and mortality; its use is increasingly widespread in the medical literature, where a very high HDI is typical of countries with more resources. Tuncalp is the first author to relate the HDI of the maternal country of origin to severe maternal outcomes such as MNM and MM with data from countries in Africa, Asia, Latin America, and the Middle East. That author describes a significant relationship between mothers from countries with medium and low HDIs; women in those countries are shown to have a risk of maternal complications that is 2-3 times higher than for women from countries with high HDIs [3].

Using the HDI of pregnant women from other countries and assessing the influence of HDI on maternal-perinatal health in our country, Spain, a previous study conducted by our team [4] observed an increased risk of adverse maternal-perinatal events in pregnant women from low-HDI countries compared to women originating from countries with higher HDIs. Similarly, Luque-Fernandez et al. [5], analysing the trend of stillbirth in Spain, showed an increased risk of stillbirth, approximately three times higher, in pregnant women from low-HDI countries. For both authors, incorporating HDI improves the characterisation of the maternal socio-economic level by introducing the HDI of the maternal country of origin and maternal educational attainment to population analysis, producing a fuller analysis compared to those studies that only include the country of origin of immigrant pregnant women.

In this study, we will consider the HDI of the place of publication (as a proxy measure like that used in the study on immigration) and determine the relationship with adverse maternal-perinatal outcomes.

The aim of this study is to conduct a systematic review of the articles published over the last decade reporting severe acute maternal morbidity. We use as a reference the HDI of the country where the study was conducted—which directly reflects the HDI of its population of pregnant women—to analyse its relationship with relevant adverse maternal-perinatal outcomes during pregnancy, childbirth, and the postpartum period, such as MNM and MM.

## Methods

### Protocol, eligibility criteria, information sources and search strategies

This review was performed according to an a-priori-designed protocol recommended for systematic reviews. PRISMA [6] and MOOSE guidelines were followed [7]. The study was registered in the PROSPERO database (registration number: CRD 42019133464). The systematic literature search was conducted in two electronic databases, PubMed/MEDLINE and EMBASE, utilising combinations of the relevant medical subjects by MeSH terms with the following keywords: “*near miss”* or “*morbidity*” and “*pregnancy*” or “*mothers*” or “pregnancy outcome”. The search period was between 17/02/2008 and 17/02/2019. A reference database (EndNote X7, Thomson Reuters) was used to incorporate all references.

The inclusion criteria were as follows:

- studies published between 17/02/2008 and 17/02/2019;
- studies conducted with humans;
- studies in English, both the abstract and the main text; and
- studies that included MNM analysis in their study population.

The exclusion criteria were as follows:

- studies with scarce information about the study population, such as country of origin, or studies investigating specific ethnic, racial, or immigrant groups;
- published articles that did not report data on MNM or those on maternal morbidity events not meeting MNM criteria according to the WHO;
- systematic reviews, expert opinions, and intervention studies without quantitative data about the MNM rate; and
- studies conducted on the same patient cohort. In these cases, we selected the most up-to-date patient cohorts and excluded secondary analysis studies on the same sample.

### Study selection

Titles and abstracts of the search results were screened by two researchers independently (SGTL and FAV). If the title and abstract did not provide useful information for the review or was irrelevant, the articles were eliminated from the analysis. Potentially eligible studies were assessed in full-text format. Any disagreement on the eligibility of studies was resolved through discussion and joint assessment until consensus was reached between the two researchers.

### Data collection and data items

Data were extracted using an appraised extraction form. Each reviewer collected the data independently, and discrepancies between them were resolved by the two authors checking the study against the form. The review authors were not blinded to the journal or author details. Extracted data included the name of the first author and year of publication, first and last year of the study, study period, country or countries where the study was conducted, HDI group to which the study country belongs, and the HDI score of the study country.

The HDI is a summary measure of a country’s average level of achievement in the following major dimensions of human development: living a long and healthy life, being educated, and having a decent standard of living. Life expectancy serves as an indicator of the health dimension; standard of living is measured in terms of gross national income per capita; and education level is evaluated as the average number of years of schooling among adults aged twenty-five years and older and expected number of years of schooling among children [8].

A country obtains a higher HDI score when its population has a higher life expectancy, education level, and gross national income (GNI) per capita; these scores are reported within the annual Human Development Report published by the United Nations Development Programme (UNDP) [9]. The UNDP divides countries into four broad categories of human development: group 1 (very high HDI), group 2 (high HDI), group 3 (medium HDI), and group 4 (low HDI) based on the numerical score obtained, with a minimum of 0 and a maximum of 1.

Other maternal-perinatal variables included in the study were type of study (single- or multi-centre), study design, total number of live births (LBs), number of MNM events in the study, rate of MNM/1000 LBs, number of maternal mortality events, rate of MM/100,000 LBs, percentage of MNM due to haemorrhage, percentage of MNM due to hypertensive disorders of pregnancy, percentage of MNM due to sepsis, percentage of MNM due to other causes, MNM in the immigrant population, MNM by ethnic group, maternal age at MNM, percentage of primiparous mothers in the MNM group, parity in MNM, percentage of births <37 weeks gestation in the MNM group, caesarean section rate in the MNM group, and neonatal near miss.

In the case of multicountry studies, the average HDI score given by the HDI scores of all included countries was calculated.

After data collection, the data were ordered according to the publication year.

### Risk of bias assessment and statistical analysis

The risk of bias was assessed independently by both authors, who determined the adequacy of compliance with the inclusion criteria. The items assessed were correct description of MNM cases, complete reporting of proportion and type of near miss in the case group, and adequate description of the country or countries where the study was carried out. We tried to choose strict eligibility criteria to achieve a good number of studies that were as homogeneous as possible and thereby extract concrete and valid conclusions.

The quality of the evidence of the studies included was assessed according to the Grade of Evidence Working Group Criteria [10].

Statistical analyses were carried out using STATA, version 13.1 (Stata Corp., College Station, TX, USA) in its default settings. The results are expressed as rates (%) for dichotomous variables, and we calculated 95% confidence intervals (95% CIs). We tried to perform a quantitative synthesis with pooled relative risks and 95% confidence intervals (95% CI), but a meta-analysis was not feasible given the lack of a control group and the heterogeneity of the available studies.

## Results

**Fig. 1:**
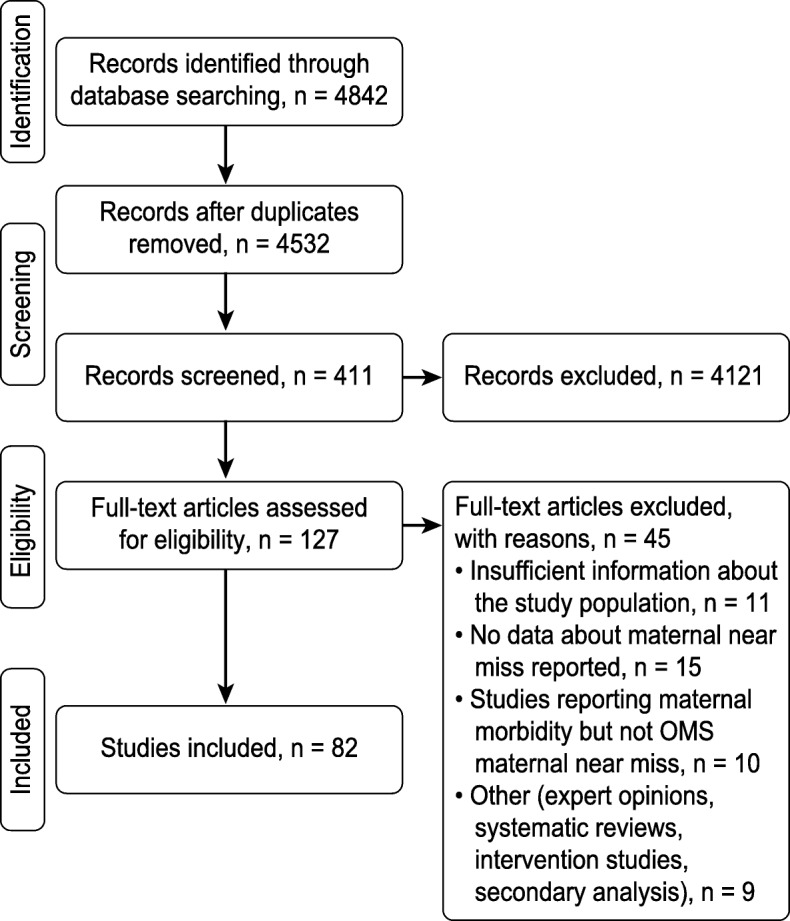
Work flow process

Figure 1 describes the workflow process. As shown, the initial search identified 4842 articles in the databases. After screening and applying the eligibility and exclusion criteria in the final phase of the records, eighty-two articles were selected. A total of 3,699,697 LBs, 37,191 near miss cases and 4029 mortality cases were reported, representing the population analysed in this systematic review.

Table 1 describes the results obtained in each study for the different variables analysed in the review. Over 90% of the studies were led by different authors; among those who led in publishing, the author who published the most studies in the period included in this analysis of MNM was Jayaratnam, with four. Of all the articles, sixty-two (75.6%) have been published since 2014, and the study by Okusanya et al. [53] (reference) included the longest period of data collection, at twenty years. Over 70% of the studies had a follow-up design with retrospective data collection/analysis.

**Table 1:** Summary of all the studies included in the review with their results

Authors	Publication Year	First Year	Last Year	Period Years	Country	HDI Group	HDI score	Study Type	Study Design	Total live births	MNM cases	MNM rate	MM cases	MM rate	MNM Haemorrhage %	MNM Hypertension %	MNM Sepsis %	MNM Others %	MNM immigrants	MNM ethnicity	MNM Maternal age	G1 in MNM %	Parity in MNM	GA < 37 weeks in MNM %	Caesarean rate in MNM %	Neonatal near miss
Adisasmita et al. [11]	2008	2003	2004	1	Indonesia	3	0.694	multi-centre	Retrospective longitudinal	5669	763	134.6	127	2240	40.6	32.3	NR	16.3	NR	NR	NR	NR	NR	NR	NR	NR
Driul et al. [12]	2008	1998	2008	10	Italy	1	0.88	single-centre	Retrospective longitudinal	18936	95	5.0	1	5.4	NR	NR	NR	NR	NR	NR	NR	NR		NR	NR	NR
Roost et al. [13]	2009	2006	2007	1	Bolivia	3	0.693	single-centre	Retrospective longitudinal	8136	401	49.3	15	187.0	48	46	NR	NR	NR	NR	NR	NR		NR	NR	NR
Almerie et al. [14]	2010	2006	2008	2	Syria	4	0.536	single-centre	Retrospective case-control	28025	901	32.1	15	54.8	34	52	2.8	NR	NR	NR	Mean 28.4 years	28	P0 28%; P1-3 40.8%; P≥4 (31.1%) in NM	NR	54%	NR
Shrestha et al. [15]	2010	2009	2009	1	Nepal	3	0.574	single-centre	Retrospective longitudinal	1562	36	23.0	5	324.0	41.6	27.7	19.4	8.3	NR	NR	Mean 27 years	30.5	G1 NM= 30.5%	NR	NR	2.77% shoulder dystocia
Souza et al. [16]	2010	2005	2005	1	Multicountry		0.745	multi-centre	Retrospective longitudinal	97095	2964	34.0	25	26	NR	NR	NR	NR	NR	NR	NR	NR		NR	NR	NR
Ali et al. [17]	2011	2008	2010	2	Sudan	4	0.502	single-centre	Retrospective cohort	9578	205	21.4	41	432.0	40.8	18	21.5	NR	NR	NR	Mean 25.5 years	NR	Mean 3.01 in NM	NR	NR	NR
Amaral et al. [18]	2011	2005	2005	1	Brazil	2	0.759	single-centre	Retrospective longitudinal	4491	95	21.1	4	89	17.9	57.8	14.3	17.8	NR	NR	NR	NR		NR	NR	60 perinatal deaths
Donati et al. [19]	2011	2004	2005	1	Italy	1	0.88	multi-centre	Retrospective longitudinal	539382	1259	2.3	NR	NR	40	29	3	25	Immigrants OR 3	NR	≥ 35 years 2.8/1000	NR	Not specified	NR	70%	NR
Jayaratnam et al. [20]	2011	2009	2010	1	Australia	1	0.939	single-centre	Prospective longitudinal	NR	17	6.0	NR	NR	40	12	NR	NR	NR	NR	NR	NR	Not specified	NR	NR	NR
Kaye et al. [21]	2011	2010	2010	1	Uganda	4	0.516	single-centre	Prospective cohort	140	21	150.0	NR	NR	NR	NR	NR	NR	NR	NR	NR	NR	Mean 3.3	NR	67.90%	NR
Lobato et al. [22]	2012	2008	2008	1	Brazil	2	0.759	single-centre	Retrospective review	1163	27	23.2	NR	NR	4	80	NR	NR	NR	NR	NR	NR	Not specified	NR	NR	NR
Souza et al. [23]	2012	2009	2010	1	Brazil	2	0.759	multi-centre	Retrospective longitudinal	82388	770	9.3	140	170.0	NR	NR	NR	NR	NR	NR	NR	NR	Not specified	NR	NR	NR
Adeoye et al. [24]	2013	2006	2007	1	Nigeria	4	0.532	multi-centre	Prospective case-control	375	75	200.0	NR	NR	45.3	37.3	18.6	NR	NR	NR	>40 years 5.3%	NR	1-2 (61.3%); 3-4 (25.3%); 5 or more (13.4%) in NM	NR	NR	NR
Jabir et al. [25]	2013	2010	2010	1	Iraq	3	0.685	multi-centre	Cross-sectional	25472	129	5.1	16	62.8	65.9	21	NR	NR	NR	NR	NR	NR	Not specified	NR	67.83%	NR
Karolinski et al. [26]	2013	2008	2009	1	Argentina	1	0.825	multi-centre	Cross-sectional	65033	518	8.0	34	52.3	36.7	31.1	4.4	15.3	NR	NR	>35 years in 21.8%, <20 years in 16.1%	26.6	26.6% P0; 37.5% >P3 in NM	NR	80.1	NR
Nelissen et al. [27]	2013	2009	2011	2	Tanzania	4	0.538	single-centre	Prospective longitudinal	9136	216	23.6	32	350.3	NR	NR	NR	NR	NR	NR	NR	NR	Not specified	NR	NR	NR
Roopa et al. [28]	2013	2011	2012	1	India	3	0.64	single-centre	Retrospective longitudinal	7390	131	17.8	23	313.0	44.2	23.6	16	NR	NR	NR	NR	58		NR	NR	NR
Shen et al. [29]	2013	2008	2012	4	China	2	0.752	single-centre	Retrospective longitudinal	18104	69	3.8	3	16.0	36.1	31.7	NR	NR	aOR in Immigrants 2.34 (95% CI, 0.45–24.9)	NR	Mean 28 ± 5 years	76.8	G1 76.8% in NM	NR	89.9	40% admission to neonatal ICU
Tuncalp et al. [3]	2013	2010	2011	1	Multicountry		0.649	multi-centre	Retrospective longitudinal	314623	1667	5.3	360	114.4	NR	NR	NR	NR	MNM by groups: 0.8% HDI 1-2, 0.5% HDI 3, 1.1% HDI 4	NR	≥35 years 10.6%	NR	G1 37.3% of the total	NR	NR	NR
Wahlberg et al. [30]	2013	1998	2007	9	Sweden	1	0.933	multi-centre	Retrospective longitudinal	914474	2655	2.9	22	2.4	NR	NR	NR	NR	Specified by groups of origin	NR	Specified by groups of origin	NR	Specified by groups of origin	NR	NR	NR
Abalos et al. [31]	2014	2004	2008	4	Multicountry		0.655	multi-centre	Cross-sectional	313030	1227	3.9	204	65.2	NR	NR	NR	NR	NR	NR	NR	NR	P2-4 51.9% in no preeclampsia group; 45.6 % in preeclampsia; P1 61.6% in eclampsia group	NR	NR	NR
David et al. [32]	2014	2008	2008	1	Mozambique	4	0.437	multi-centre	Cross-sectional	27916	564	20.2	71	254.0	58	35.5	3.9	NR	NR	NR	14-19 (23.6%), 20-24 (27%), 25-29 (26.2%), 30-34 (16.7%), ≥35 (6.6%)	33.9	0 (33.9%); 1 (20.47%); 2-4 (40.6%); ≥5 (4.8%) in NM	NR	56.6	NR
Galvao et al. [33]	2014	2011	2012	1	Brazil	2	0.759	multi-centre	Cross-sectional/Nested case-control	16243	77	4.7	NR	NR	NR	NR	NR	NR	NR	84.4% non white; 15.6% white	< 35 years 73.9%; ≥35 years 26.1%	NR	Not specified	NR	74.5	NR
Litorp et al. [34]	2014	2012	2012	1	Tanzania	4	0.538	multi-centre	Cross-sectional	13121	467	35.6	77	587.0	13	42	NR	NR	NR	NR	Mean 26 years	43	P0 (43%); 1-4 (50%); >4(3.9%); in NM	NR	35	NR
Luexay et al. [35]	2014	2011	2011	1	Laos	3	0.601	multi-centre	Retrospective longitudinal	1215	11	9.1	2	178.0	NR	NR	NR	NR	NR	Lao (70.6%); tribes (18.3%)	Mean 24.4 years	43	G1 43% of the total	12.8	NR	NR
Lumbiganon et al. [36]	2014	2015	2011	1	Multicountry		-	multi-centre	Cross-sectional	314623	2365	7.5	NR	NR	NR	8.1	28.1	NR	NR	NR	NR	NR	Not specified	NR	NR	NR
Mazhar et al. [37]	2014	2011	2011	1	Pakistan	4	0.562	multi-centre	Retrospective longitudinal	13175	94	7.1	38	299.0	48.5	25.8	NR	NR	NR	NR	20-40 years 96.2 %	37	G1 37% in NM	47	49	NR
Pacheco et al. [38]	2014	2011	2011	1	Brazil	2	0.759	single-centre	Retrospective longitudinal	2291	24	10.5	3	130.9	NR	NR	NR	NR	NR	NR	NR	NR	Not specified	NR	29.7	NR
Pandey et al. [39]	2014	2011	2012	1	India	3	0.64	single-centre	Retrospective longitudinal	6357	633	120.0	247	4684.0	45.6	24.2	7.5	8.7	NR	NR	NR	NR		NR	NR	NR
Rocha Filho et al. [40]	2014	2009	2010	1	Brazil	2	0.759	multi-centre	Retrospective longitudinal	82144	770	9.4	140	170.4	43.5	NR	NR	56.5	NR	43.1% white; 56.9% non white	≥40 years 7%	38.9	G1 38.9% in NM	72.3	89.5	NR
Assarag et al. [41]	2015	2012	2012	1	Morocco	3	0.667	multi-centre	Retrospective case-control	299	80	267.6	NR	NR	39	45	10	5	NR	NR	Mean 29.2 years	50	P1 (50%); 2-3 (39%); ≥4 (11%) in NM	NR	66	NR
Bashour et al. [42]	2015	2011	2015	4	Multicountry (Egypt, Lebanon, Palestine and Syria)		0.616	multi-centre	Cross-sectional	9063	71	7.8	6	66.2	100	15.4	NR	30.9	NR	NR	NR	NR	(Egypt 40.7%) 3-4; (Lebanon 60%) 0; (Palestine 43.8%) >5; (Syria 27.8%) 0, 1-2, 3-4	NR	Egypt 65.6%; Lebanon 100%; Palestine 50%; Syria 61.1%	NR
Cecatti et al. [43]	2015	2009	2010	1	Brazil	2	0.759	multi-centre	Cross-sectional	9555	770	80.6	16	170.0	40.5	45.3	5.7	NR	NR	NR	NR	NR	Not specified	NR	NR	NR
Hassan et al. [44]	2015	2011	2012	1	Palestine		-	single-centre	Prospective longitudinal	1558	15	9.6	NR	NR	16.4	4.2	2.5	26.9	NR	NR	NR	16.2	G1= 253 (16.2%) of the total	NR	2420.00%	0.6% admision UCI, 14 perinatal deaths
Kulkarni et al. [45]	2015	2012	2013	1	India	3	0.64	multi-centre	Prospective longitudinal	19176	884	46.1	94	490.2	7.7	53.4	NR	NR	NR	NR	Mean 25.8 years	41	41% G1 in NM	NR	NR	NR
Madeiro et al. [46]	2015	2012	2013	1	Brazil	2	0.759	single-centre	Cross-sectional / Prospective longitudinal	5841	56	9.6	10	171.2	100	86.1	NR	NR	NR	NR	<20 years 25.8%	NR	≥4 13.6% in NM	54.8	87.5	NR
Naderi et al. [47]	2015	2013	2013	1	Iran	2	0.798	multi-centre	Retrospective longitudinal	19908	501	25.2	2	10	46.1	31.9	NR	15.2	NR	NR	NR	41.5		NR	54.2	NR
Oladapo et al. [48]	2015	2012	2013	1	Nigeria	4	0.532	multi-centre	Prospective longitudinal	91724	1451	15.8	998	1088.0	49	20.5	2.5	NR	NR	NR	NR	NR	Not specified	NR	NR	perinatal deaths 60.5/1000 live births
Oliveira et al. [49]	2015	2006	2007	1	Brazil	2	0.759	single-centre	Retrospective longitudinal	19940	255	12.8	56	280.8	53.7	62.7	NR	NR	NR	57.3% mixed, 17.6% white, 7.1% black	≥35 years 11.8%	44.7	G1 44.7% in NM	54.5	76.4	NR
Rulisa et al. [50]	2015	2011	2012	1	Rwanda	4	0.524	single-centre	Retrospective longitudinal	1739	192	110.4	50	2875.2	19.3	28.6	30.2	NR	NR	NR	≥35 years 15.6%	NR	Not specified	45	45.5	NR
Sangeeta et al. [51]	2015	2012	2013	1	India	3	0.64	single-centre	Retrospective longitudinal	6892	27	4.0	8	116	40.7	26	7.4	NR	NR	NR	NR	NR		NR	NR	NR
Soma-Pillay et al. [52]	2015	2013	2014	1	South Africa	3	0.699	multi-centre	Retrospective longitudinal	26614	136	5.1	19	71.4	37.5	32.4	10.3	NR	NR	NR	NR	29		NR	NR	NR
Okusanya et al. [53]	2016	1993	2013	20	Nigeria	4	0.532	single-centre	Retrospective cross-sectional	30553	116	3.8	NR	NR	NR	NR	NR	NR	NR	NR	20-24 n=3; 25-29 n=31; 30-34 n=40; 35-39 n=33; 40-44 n=9	NR	0 n=6; 1 n=20 ; 2 n=27; 3 n=35; 4 n=14; 5 n=14	NR	NR	NR
de Mucio et al. [54]	2016	2013	2013	1	Latin America (12 countries)		0.723	multi-centre	Cross-sectional	3196	37	11.6	NR	NR	NR	NR	NR	NR	NR	NR	NR	NR	Not specified	13.3	NR	NR
Domingues et al. [55]	2016	2011	2012	1	Brazil	2	0.759	multi-centre	Retrospective case-control	23984	244	10.2	NR	NR	NR	NR	NR	NR	NR	56.1% mixed; 33.8% white; 8.6% black; 1.1% asian; 0.4% indigenous of the total	NR	46.9	P0 46.9%; P1 29.4%; 2-3 18.8%; >4 4.9%	NR	43.7	NR
El Ghardallou et al. [56]	2016	2012	2012	1	Tunisia	2	0.735	single-centre	Retrospective longitudinal	9957	58	5.8	1	10.0	74.1	20.7	NR	25.9	NR	NR	Mean 32 ± 5.2 years, >39 years 12.1%	36.2	G1= 36.2% in NM	NR	66.7	15.4% neonatal death, 48.5% (n=16) ICU admission
Jayaratnam et al. [57]	2016	2014	2015	1	Australia	1	0.939	single-centre	Prospective longitudinal	2080	10	4.8	NR	4.8	NR	NR	NR	NR	NR	NR	NR	NR	Not specified	NR	NR	No
Kalisa et al. [58]	2016	2014	2014	1	Rwanda	4	0.524	single-centre	Prospective cohort	3979	86	21.6	13	325.0	57	31.4	NR	NR	NR	NR	NR	NR	Not specified	NR	43	No
Lima et al. [59]	2016	2009	2010	1	Brazil	2	0.759	multi-centre	Retrospective longitudinal	4617	50	10.8	10	216	NR	NR	NR	NR	NR	NR	NR	54.3		NR	NR	NR
Mohammadi et al. [60]	2016	2012	2014	2	Iran	2	0.798	multi-centre	Retrospective case-control	12965	82	6.3	12	92.6	35	32	7	NR	NR	NR	≥35 years n=124	23	G1 n=495 (23% G1 in NM)	48	81	204 perinatal deaths
Nakimuli et al. [61]	2016	2013	2014	1	Uganda	4	0.516	multi-centre	Prospective cohort	NR	695	8.4	130	503.0	26.5	22	11.8	NR	NR	NR	≥25 years 55.7%	26.5	G1 n=184 (26.5%) of NM	NR	78%	NR
Nansubuga et al. [62]	2016	2013	2013	1	Uganda	4	0.516	single-centre	Retrospective longitudinal	1557	434	278.7	NR	NR	55	0.2	3.5	4.1	NR	NR	NR	NR	Not specified	NR	NR	NR
Norhayati et al. [63]	2016	2014	2014	1	Malaysia	2	0.802	multi-centre	Retrospective longitudinal	21579	47	2.2	2	9.3	80.9	21.3	NR	38.3	NR	NR	Mean 33.2(6.03) years, >35years 42.6%	NR	Not specified	NR	63.80%	19.1% perinatal death , 63.2% admitted to neonatal ICU
Parmar et al. [64]	2016	2012	2012	1	India	3	0.64	single-centre	Retrospective longitudinal	1929	46	23.9	18	933.0	NR	NR	NR	NR	NR	NR	NR	NR		42	NR	39% perinatal death
Rathod et al. [65]	2016	2011	2013	2	India	3	0.64	multi-centre	Retrospective longitudinal	21992	161	7.6	66	300	26.7	11.8	11.5	NR	NR	NR	NR	NR		NR	NR	NR
Tanimia et al. [66]	2016	2012	2013	1	Papua New Guinea	4	0.544	single-centre	Prospective longitudinal	13338	122	9.1	9	67.5	38	32	7.4	NR	NR	NR	NR	NR	NR	NR	NR	NR
Bolnga et al. [67]	2017	2014	2016	2	Papua New Guinea	4	0.544	single-centre	Prospective longitudinal	6019	153	25.4	10	166.0	42.5	22.2	16.3	3.3	NR	NR	NR	NR	NR	NR	26.80%	NR
Goldenberg et al. [68]	2017	2014	2016	2	Multicountry (Congo, Guatemala, India, Kenia, Pakistan and Zambia)		0.593	multi-centre	Prospective longitudinal	122707	4866	39.7	190	155.0	79	42	75	NR	NR	NR	NR	NR	NR	NR	NR	NR
Herklots et al. [69]	2017	2016	2016	1	Tanzania	4	0.538	single-centre	Cross-sectional	4125	37	6.7	28	678.8	29.7	24.3	10.8	2.7	NR	NR	<20 years 12.3%; 20-35 years 66.2%; >35 years 21.5%	20	P0 20%; P1-4 60%; P>4 20%	NR	63	NR
Khan et al. [70]	2017	2009	2011	2	India	3	0.64	single-centre	Retrospective cross-sectional	20556	302	14.7	67	325.0	63.6	20.5	2.6	NR	NR	NR	Mean 26.7 years	36.4	G1 (36.4%); G2-3 (50%); G4-6 (13.6%)	NR	64.2	NR
Kiruja et al. [71]	2017	2015	2015	1	Somalia	4	-	single-centre	Retrospective longitudinal	1385	120	86.6	18	1328.0	36.7	55	2.5	1.7	NR	NR	Mean 29.5 years	2.5	≥ 7 (29.2%); 5-6 (10.8%); 2-4 (29.2%); 1 (28.3%); 0 (2.5%)	NR	NR	21.7% perinatal death
Liyew et al. [72]	2017	2015	2016	1	Ethiopia	4	0.463	multi-centre	Cross-sectional	29697	238	8.0	NR	NR	38	53	1	NR	NR	NR	NR	NR	NR	NR	NR	NR
Mawarti et al. [73]	2017	2011	2012	1	Indonesia	3	0.694	single-centre	Retrospective longitudinal	3300	86	26.0	29	879	5.81	95	4.5	NR	NR	NR	NR	50	NR	NR	NR	NR
Mbachu et al. [74]	2017	2015	2015	1	Nigeria	4	0.532	single-centre	Retrospective longitudinal	262	52	198.5	5	1908.0	24.6	28.1	1.8	NR	NR	NR	NR	NR	NR	NR	NR	NR
Mekango et al. [75]	2017	2016	2016	1	Ethiopia	4	0.463	multi-centre	Retrospective longitudinal	308	103	334.4	NR	NR	44.7	38.8	9.7	NR	NR	NR	≥40 years n=88	NR	G1 N=5	54.4	NR	NR
Sayinzoga et al. [76]	2017	2016	2016	1	Rwanda	4	0.524	multi-centre	Prospective case-control	5577	201	36.0	13	233.1	22.9	8.5	7.5	5	NR	NR	≥35 years 60%	60	G1 60%	34	52	46.1% perinatal death
Witteveen et al. [77]	2017				Multicountry (Netherlands, Tanzania, Malawi)		0.648	multi-centre	Prospective cohort	NR	2308	NR	126	NR	NR	NR	NR	NR	MNM% specified by country of origin	NR	Specified by country	NR	Specified by country	NR	NR	NR
Awowole et al. [78]	2018	2007	2016	9	Nigeria	4	0.532	single-centre	Retrospective longitudinal	11242	43	3.8	NR	NR	18	40	12	NR	NR	NR	Mean 29.2 years	NR	Mean 2	NR	NR	NR
Benimana et al. [79]	2018	2015	2015	1	Rwanda	4	0.524	single-centre	Retrospective longitudinal	NR	98	NR	NR	NR	23.1	21.5	27.3	NR	NR	NR	16-24years (28.9%); 25-34 years (52.1%); ≥35 years (19%)	17.4	0 (17.4%); 1-2 (53.7%); ≥3 (28.9%)	NR	NR	NR
Chikadaya et al. [80]	2018	2016	2016	1	Zimbabwe	4	0.535	single-centre	Prospective longitudinal	11871	110	9.3	13	109.5	31.8	28.2	NR	20	NR	NR	NR	NR	NR	NR	NR	NR
Iwuh et al. [81]	2018	2014	2014	1	South Africa	3	0.699	multi-centre	Retrospective longitudinal	19222	112	5.8	13	67.6	33.9	44.6	11.6	NR	NR	NR	<18 years 3.6%; 18-34 years 84.8%; ≥35 years 11.6%	41.1	P0 41.1%; P1-4 58%; P5 0.9%	NR	NR	NR
Jayaratnam et al. [82]	2018	2014	2015	1	Australia	1	0.939	single-centre	Prospective longitudinal	2773	19	7.0	NR	NR	NR	NR	NR	NR	NR	NR	NR	NR	NR	NR	NR	NR
Liyew et al. [83]	2018	2015	2016	1	Ethiopia	4	0.463	multi-centre	Prospective cohort	828	207	250.0	NR	NR	NR	NR	NR	NR	NR	NR	NR	NR	P 0-2 (79.2%); P3-4 (15.5%); P>5 (5.3%)	40.6	NR	29.5% perinatal death
Oliveira Neto et al. [84]	2018	2013	2015	2	Brazil	2	0.759	single-centre	Retrospective longitudinal	8065	60	7.4	5	62	64.5	25.8	6.5	NR	NR	NR	>35 years 75%	NR	NR	NR	74	NR
Tura et al. [85]	2018	2016	2017	1	Ethiopia	4	0.463	single-centre	Retrospective longitudinal	7404	594	80.2	28	378	36	45.6	21.2	NR	NR	NR	NR	NR	NR		NR	NR
Woldeyes et al. [86]	2018	2015	2015	1	Ethiopia	4	0.463	single-centre	Retrospective longitudinal	2737	138	50.4	24	877.0	22.5	21	10.1	5.8	NR	NR	NR	41.6	NR	NR	25.7	NR
Yang et al. [87]	2018	2012	2015	3	China	2	0.752	single-centre	Retrospective longitudinal	14105	265	18.8	10	70.9	36.9	49	NR	NR	NR	NR	≥35 years 2.54%	22.3	G1-2 2.33%	5.36	NR	35 perinatal deaths
Herklots et al. [88]	2019	2017	2018	1	Tanzania	4	0.538	single-centre	Prospective longitudinal	26842	256	9.5	79	294	NR	NR	NR	NR	NR	NR	NR	NR	NR	NR	NR	NR
Jayaratnam et al. [89]	2019	2015	2016	1	Timor	3	0.625	single-centre	Prospective longitudinal	4529	39	8.0	30	662	25	25	NR	NR	NR	NR	NR	50	NR	NR	NR	NR
Oppong et al. [90]	2019	2015	2015	1	Ghana	3	0.592	multi-centre	Retrospective longitudinal	8433	288	34.2	62	735	12.2	41	11.1	NR	NR	NR	NR	NR	NR	NR	NR	NR
Zanardi et al. [91]	2019	2009	2010	1	Brazil	2	0.759	multi-centre	Retrospective longitudinal	82388	624	7.6	113	137.1	NR	NR	NR	NR	NR	NR	NR	37	NR	63%	73.9	14.2% perinatal death

Looking at single-country studies, over thirty-three countries were represented, and seven studies were conducted with populations from several countries; Brazil published more studies than any other country, with thirteen (15.4%), followed by India, with six (7.1%), and Nigeria and Ethiopia, with five each (6%). Regarding the number of studies classified by HDI group, seven belonged to group 1, nineteen to group 2, eighteen to group 3, and twenty-nine to group 4. In only three studies, the HDI score could not be obtained because of the lack of data provided regarding the study country.

Regarding the MM rate, the median was 175 deaths per 100,000/LBs, with six studies reporting a rate above 1000; in relation to the MNM rate, the median was 11 events per 1000 LBs, with nine studies reporting a rate above 100. Regarding MNM, the average of the overall percentage of publications reported the cause to be haemorrhage (38.5%), hypertensive disorders of pregnancy (34.2%), sepsis (7.5%), and other causes (20.9%).

In relation to gestational data, the mean percentage of primiparous women in the total cases of MNM published was 37%. The mean percentage of premature births in the MNM cases was 38%. The mean percentage of caesarean sections in the MNM cases reported in the twenty-eight articles that reported these data was 57.2%.

Of all the articles included in the review, only sixteen presented data on adverse neonatal outcomes; the most commonly described complication was perinatal death, reported in twelve articles.

Finally, 4/82 articles referred to the differential analysis of near-miss ratios in immigrants, and 16/82 provided data on perinatal mortality or morbidity (near miss) in their results.

**Fig. 2:**
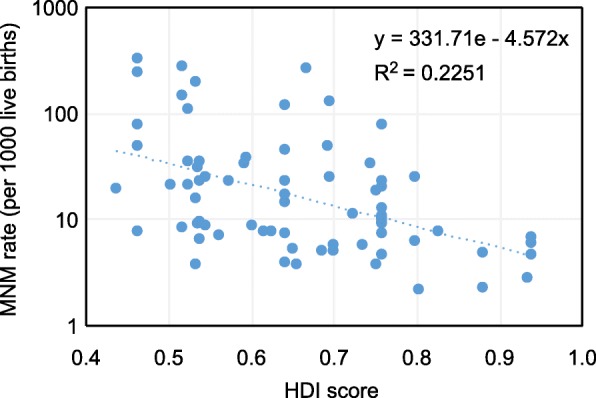
Relationship between HDI score and MNM rate

**Fig. 3:**
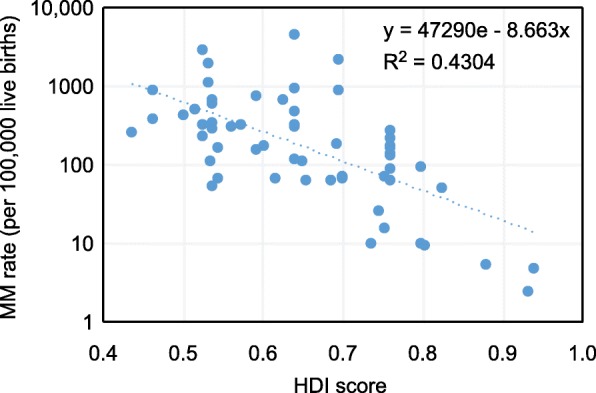
Relationship between HDI score and MM rate

Figures 2 and 3 show the exponential trend relationship between the HDI score of the study population and the MNM and MM rates. In both, an inversely proportional relationship between the two variables was shown; higher MNM rates and higher MM rates were observed for study countries with lower HDI scores, significantly in both cases:

- Average rate of MNM/country = 331.71e^-4.572country HDI^ per 1000 live births (R^2^ = 0.2251; *p* = 0.001)
- Average rate of MM/country = 47290e^-8.663country HDI^ n per 100,000 live births (R^2^ = 0.4304; *p* = 0.038)

In addition, to provide more detail in these figures, Tables 2 and 3 show the MNM and MM rates, respectively, weighted by the number of LBs according to the HDI group of the study population. The articles whose study population belonged to HDI group 1 showed the lowest MNM and MM rates compared to the rest of the groups. Those whose study population belonged to HDI group 3 had the highest MNM rate, 7.6 times higher than that of HDI group 1. Studies whose population was classified as HDI group 4 had the highest MM rate, 98.4 times higher than that of HDI group 1. It should be noted in these tables that the MNM rate for group 4 was lower than that for HDI group 3.

**Table 2:** MNM rate weighted by the number of LBs according to the HDI group

HDI group	Sum of MNM	Sum of livebirths	MNM rate per 1000 livebirths
1	4556	1542678	2.95
2	4844	439728	11.01
3	4265	188743	22.59
4	7196	352653	20.40
Total	20861	2523802	8.26

**Table 3:** MM rate weighted by the number of LBs according to the HDI group

HDI group	Sum of MM	Sum of livebirths	MM rate per 100,000 live births
1	57	998443	5.7
2	527	398338	132.4
3	841	188444	446.3
4	1563	277953	562.2
Total	2988	1863178	160.4

**Fig. 4:**
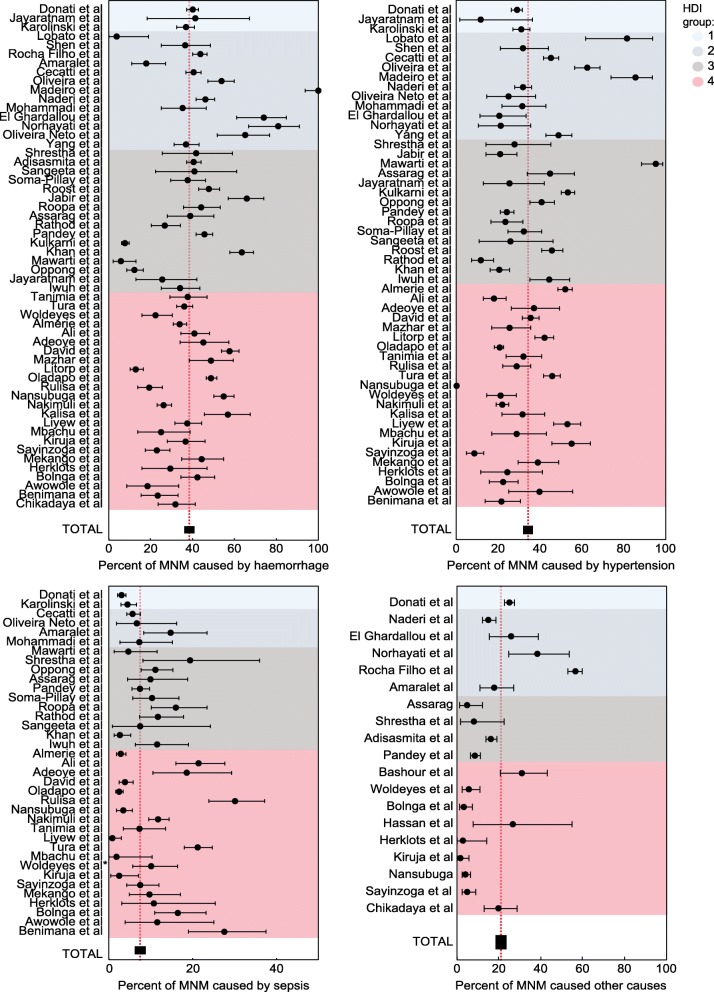
Proportion of each cause of MNM published according to HDI group

The proportion of each cause of MNM published in each study is shown in Figure 4. This same figure reflects the overall proportions of each type of MNM. The most common cause of MNM in the set of studies selected in this review was haemorrhage, occurring in 38.5% (95% CI, 37.7-39.2) of all cases.

Concerning haemorrhagic causes of MNM, the study by Lobato et al. [22] reported the lowest proportion of this complication, with 3.7%, compared to the study by Madeiro et al. [46], which reported the highest percentage of haemorrhagic causes of MNM, 100% of total cases in their sample.

Regarding hypertensive disorders as a cause of MNM, the studies by Lobato et al. [22], Madeiro et al. [46], and Mawarti et al. [73] predominantly include populations of pregnant women from countries in HDI groups 2 and 3, with proportions of MNM greater than 80% out of all cases in their respective samples.

Overall, the less common cause of MNM was infection/sepsis, at 7.5%, although the studies by Rulisa et al. [50] and Benimana et al. [79] observed this cause to be responsible for 30.2% and 27.6%, respectively, of total MNM cases. Both studies were conducted in countries belonging to HDI group 4. A total of 83.7% of studies that reported infectious causes of MNM were conducted in countries classified as HDI groups 3 and 4.

## Discussion

This systematic review of the literature selected eighty-two studies that included over three million live births, over 37,000 MNM cases, and just over 4,000 MM events over the past eleven years, representing over fifty countries.

To our knowledge, this is the most up-to-date review of MNM as an adverse perinatal outcome, and the only one in which the country of origin of the study population has been analysed. In addition, it is the first review that analyses these results in relation to the HDI of each country of publication.

As shown in Table 1, increasingly more studies are publishing MNM results as an indicator for monitoring the quality of maternal health and maternal care. These data will be a valuable contribution to taking necessary action to improve the quality of maternal care.

### MNM as an analysis variable of maternal morbidity and mortality and the importance of the country of origin

Despite the differences in MM between countries, these events are increasingly infrequent and related to an LB rate on the order of 100,000. As stated above, MNM data collection is increasingly necessary; most of the studies included have been published since 2014, showing the growing interest in considering this variable.

Brazil published the most studies in this period, followed by India, Nigeria and Ethiopia; most studies were published in low-HDI countries, leading to publication bias because, as this study shows, cases of severe maternal morbidity are more prevalent in more disadvantaged countries.

As highlighted in Table 1, only four studies underline the relationship between MNM and migration when analysing maternal origin, where perinatal outcomes were more unfavourable in immigrant groups. However, many studies analysed this variable for MM. In a systematic review that included thirteen studies involving over forty-two million women and 4995 maternal deaths, immigrant women had twice the risk of this complication over native women in Western Europe [92].

As in the results obtained in those four studies regarding both MNM and MM, our results highlight a significant relationship between the HDI of the place of publication and adverse maternal-perinatal outcomes. These results are in line with previous studies by Tuncalp et al. [3] and Luque-Fernandez et al. [5] and those reported previously by our team.

These studies highlight the importance of classifying maternal risk by considering not only economic data but also other relevant aspects of human development and capacity for survival in each country, or, in the case of immigrants, their country of origin, specifically in the case of pregnant women from low-income countries where monitoring of pregnancy and childbirth occurs in their countries of origin and when a pregnant woman becomes an immigrant in a country with higher resources. Wahlberg et al. [30] observed, in a study conducted in Sweden that included 914,474 births and 2655 MNM cases, that women from low-income countries had a significant 2.3 times greater risk than native women of suffering from severe morbidity events. This study revealed some hypotheses about plausible mechanisms by which this relationship occurred, such as a breach of previous social networks among immigrant women, low socio-economic status, poor access to health and prenatal care, and communication problems resulting from suboptimal language acquisition.

Urquia et al. [93] analysed 1,252,543 births in Ontario hospitals between 2002 and 2012 and observed heterogeneity that included severe maternal morbidity rates according to the world regions of origin of pregnant women. Overall, they found no significant differences in the risk of such pregnancy complications between native and immigrant women; however, in women from East Asia, such as Vietnam and the Philippines, an increased risk of severe maternal morbidity was observed among these patients in Canadian hospitals.

Finally, it is necessary to highlight the data from Table 1, which show that only a minority of the authors reported maternal morbidity data, such as MNM**,** and neonatal morbidity results. Less than 20% of these publications considered adverse perinatal outcomes in newborns, reporting neonatal mortality as the most common complication but poorly describing very important information such as pH at birth, Apgar score, need for neonatal resuscitation manoeuvres, or admission to the neonatal intensive care unit.

### Main findings

The present study shows that MNM and MM rates have a significant relationship with maternal country of origin. Specifically, the HDI of the maternal country of origin where the different studies were conducted was significantly related to MNM and MM rates. Thus, we have observed that the lower the HDI score of the maternal country of origin, the greater the risk is of suffering from these 2 severe pregnancy complications.

We must emphasise that HDI group 3 had the highest MNM rate compared to the other groups even though group 4 would be expected to have the worst results for this complication. The reason for this is not explained in our review, although a possible cause could be that HDI group 4 had lower MNM ratios compared to group 3 because cases of severe morbidity in these countries more frequently caused maternal deaths. This hypothesis would explain why HDI group 4 had an overall MM rate higher than Group 3 and other groups.

Thus, the present study allows calculation of the average expected MNM ratios based on the country's HDI score, as shown in the following examples:

**Table:** 

- Average MNM rate in Sweden = 331.71e^-4.572x0.933^ = 4.69 per 1000 LBs - Average MNM rate in Brazil = 331.71e^-4.572x0.759^ = 10.38 per 1000 LBs - Average MNM rate in Uganda = 331.71e^-4.572x0.516^ = 31.54 per 1000 LBs	

In the same way, if we wanted to calculate the average expected MM rate in a country based on its HDI, we could apply the following formula presented in the results section:

**Table:** 

- Average MM rate in Sweden = 47290e^-8.663x0.933^ = 15.02 per 100,000 LBs - Average MM rate in Brazil = 47290e^-8.663x0.759^ = 67.46 per 100,000 LBs - Average MM rate in Uganda = 47290e^-8.663x0.516^ = 549.73 per 100,000 LBs	

We can observe how the MNM and MM rates increase as the HDI score of the reference country decreases. On the other hand, we see rates of these complications similar to those published by the authors of the studies included in this review. The calculation of these rates is limited by the use of a single explanatory variable such as the HDI score of the country in which the adverse event occurs in the study; therefore, we can observe differences in the results published by other authors, such as the study by Vangen et al. [94] in Norway, which presented an HDI score similar to that of Sweden and a MM rate of 7.2 per 100,000 LBs, half of what was anticipated from our equation.

Estimating these two severe adverse events of pregnancy, childbirth, and the postpartum period can be important for clinicians, enabling them to classify the risk of such events according to the place of maternal origin. Considering previous calculations, a clinician in Sweden can expect that near-miss and mortality rates for a patient attending their hospital from Uganda may be higher than those of a patient from Brazil (if we consider the rates of these countries and how to discriminate between Uganda and Brazil), even if both are immigrants. Obviously, this hypothesis must be confirmed by more studies; surely, the near-miss rate of an immigrant patient in Sweden is lower than that corresponding to their country of origin, but according to our results, it is possible that HDI can help estimate the risk with more accuracy.

The HDI simplifies and captures major socio-demographic characteristics and encompasses various aspects of human development across countries in the form of a common score, as explained above. Therefore, using the HDI, maternal origin can be categorised not only by race and ethnicity but also by income and educational level, which provide accurate information regarding poverty and inequality worldwide. According to our systematic review, the excess risk of MNM and MM seems to depend not only on the maternal birthplace but also on the region where the prenatal checkups and delivery took place, other maternal characteristics and the presence of comorbidities. Therefore, taking into account that a significant proportion of MNM and MM cases are avoidable, there should be an initiative to develop and implement epidemiological analysis systems in host countries to identify socio-demographic risk factors – such as indicators of poverty and social impairment – that have a significant impact on the perinatal outcomes of pregnant immigrant women.

This proposal to use HDI as a parameter related to morbidity and mortality rates is another step in calculating these risks by analysing other aspects than just the average income of the maternal country of origin or immigrant status. Previously, other authors showed an increased risk of severe maternal morbidity events during pregnancy, childbirth, and the postpartum period in women from low-income countries, such as those in sub-Saharan Africa and the Caribbean [95–97]. The study published by Blagoeva Atanasova et al. [98] in Spain showed a significantly increased MM risk (four times higher) in immigrant women from South American countries. Similarly, this study highlighted important inequalities in the rate of this complication depending on the place of maternal origin.

### Near-miss types by HDI group (Figure 4)

Our review showed that the most common cause of MNM was haemorrhage (38.5% of cases), followed closely by hypertensive disorders of pregnancy.

Overall, we did not observe significant differences in the proportions of MNM types according to the HDI or maternal HDI groups. Thus, although the absolute number and MNM rate are higher in low-HDI countries compared to countries with higher HDI, the proportion of causes of these maternal morbidity events does not differ substantially from one country to another for reasons that are not clear in the literature.

Published studies reflect heterogeneous results in the proportions of MNM, as in a recent multi-centre analysis published by Oppong et al. [90] conducted in Ghana with 8,433 LBs and 288 MNM cases. In this study, the most common cause of MNM was preeclampsia/eclampsia, at 41%, compared to haemorrhage, which was observed in 12.2% of cases. The identification and classification of near-miss cases were performed in this group using the WHO Maternal Near Miss Tool [23].

Tanimia et al. [66], however, in a study conducted in Papua New Guinea with 13,338 LBs and 122 near-miss cases, identified, using the same tool and WHO criteria, haemorrhage as the most common cause of maternal near miss (38%), followed by hypertensive disorders of pregnancy (32%).

The main cause of MM identified by the Global Burden of Disease (GBD) study, which conducted a global and regional review of data from 186 countries during the period of 1990–2015, was obstetric haemorrhage. Other relevant causes of MM were hypertensive disorders of pregnancy, maternal sepsis, obstructed labour, and uterine rupture [99].

There are several reasons why the proportion of MNM causes may differ from one study to another even among countries with similar socio-economic development levels as defined by the HDI. On the one hand, the method used in the collection, definition, and classification of MNM varies from one study to another in both the sources and classification systems of these pregnancy complications. There are several cases in which patients may suffer from several types of near-miss incidents, or one cause of near miss may trigger another, but these situations may not be revealed in the results of the studies included in this review. Furthermore, the description of the study population and hospitals where the conditions were treated in the various studies were not always sufficiently detailed to identify the reason why, in some studies, one cause of near miss was more prevalent than another. In this regard, the maternal HDI given by the country of origin where each study was conducted does not explain the differences found between the studies in the proportion of each type of MNM.

### Strengths of the review

This is the most recent and up-to-date systematic review that addresses the importance of characterising pregnant women by their country of origin and investigates a relevant sociodemographic variable, HDI, and its relationship with adverse events such as MNM and MM. From what has been published over the course of a decade, eighty-two articles were collected, describing results from over forty countries, including a large number of patients and maternal morbidity and mortality events.

### Limitations of the review

Several limitations are worth considering when interpreting the results of this review. However, there is a lack of uniform criteria for the identification of cases of severe obstetric morbidity or MNM. The identification of cases is complex and varies across studies. Three major criteria have been mentioned in a review conducted by the WHO [100]. The review suggested the use of organ system dysfunction-based criteria supplemented with compatible clinical markers of organ system dysfunction that are feasible for collection in the absence of higher-level amenities-based criteria for identifying all severe morbidity and investigating the cause as the most reproducible one across similar areas.

Population characteristics in case-control groups were not always well described; in several studies, relevant adjustment variables of perinatal outcomes were not used, such as maternal comorbidities, maternal age, parity, maternal body mass index (BMI), or belonging to ethnic or sociodemographic groups that are more vulnerable to pregnancy complications.

As we have described, very few studies refer to immigrant pregnant women or maternal HDI influencing adverse events during pregnancy, childbirth, and the postpartum period.

To address these limitations, Mengistu et al. [101] have recently published a protocol for the systematic review and meta-analysis of severe maternal morbidity events and MNM**,** at least in high-income countries.

Finally, we must note the limitations of the HDI. On the one hand, the population in the study country is not homogeneous with regard to origin, education level, or income; these factors are not always perfectly described in national epidemiological publications or data. On the other hand, migration flows are very diverse from one country to another depending on economic, social, political, and geographical factors; therefore, the quantity and characteristics of the immigrant population of a nation can be more or less heterogeneous even within similar territories, as in the European Union. We attempted to divide the patients into groups in a simple manner that was based on maternal HDI; additionally, we obtained as much information as we could regarding the mothers’ social situation, as indicated by their country of origin but this might not be entirely informative.

## Conclusions

In summary, this review of the literature highlights the usefulness of identifying the HDI of the maternal country of origin through the HDI of the country of publication. Based on eighty-two articles, the review includes a great variety of countries, patients, and maternal morbidity and mortality events. This variety has allowed us to study the inverse and significant relationship between maternal morbidity and mortality and the HDI of the countries included. This relationship is maintained according to the HDI groups.

The most common causes of MNM described were haemorrhage and hypertensive disorders of pregnancy and, less frequently, infectious complications and sepsis. Overall, there were no significant differences in the proportion of each cause of MNM, the HDI, and HDI groups.

### Implications for clinical practice

This study shows that the use of maternal sociodemographic variables, including the HDI, may be useful to categorise the risk of maternal morbidity and mortality. In addition to economic value, the HDI weighs education level and life expectancy – as health and social parameters of pregnant women – according to their origin. The HDI is a variable that is easily accessible and calculated, although it may have limitations influenced by other factors, for example, in the immigrant population, such as time spent in the destination country, baseline health state, or the degree of social integration and family income. More studies are needed to determine the discriminatory value of risk in the immigrant population treated in different countries.

## Data Availability

Data from this systematic review is available as supplementary material in table 1 and provided upon request.

## Abbreviations

MM: Maternal mortality
MNM: Maternal near miss
HDI: Human development index
WHO: World Health Organization
GNI: Gross national income
UNDP: United Nations Development Programme
LB: Live births
NR: Non reported
GBD: Global Burden of Disease
BMI: Body mass index
